# Workplace Stressors Associated With Burnout Among Emergency Nurses and Other Emergency Healthcare Professionals: A Convergent Parallel Approach With a Multilevel Design

**DOI:** 10.1155/jonm/7360364

**Published:** 2026-05-25

**Authors:** Lisa M. Vizer, Chao Chin Liu, Elizabeth K. Kwong, Nicholas Beresic, Karthik Adapa, Charul Haugan, Nadia E. Charguia, Lukasz M. Mazur

**Affiliations:** ^1^ Division of General Internal Medicine & Clinical Epidemiology, School of Medicine, University of North Carolina at Chapel Hill, Chapel Hill, North Carolina, USA, unc.edu; ^2^ Division of Healthcare Engineering, School of Medicine, University of North Carolina at Chapel Hill, Chapel Hill, North Carolina, USA, unc.edu; ^3^ School of Information and Library Sciences, University of North Carolina at Chapel Hill, Chapel Hill, North Carolina, USA, unc.edu; ^4^ Thurston Arthritis Center, University of North Carolina at Chapel Hill, Chapel Hill, North Carolina, USA, unc.edu; ^5^ UNC Healthcare System, Chapel Hill, North Carolina, USA, unc.edu; ^6^ Department of Psychiatry, School of Medicine, University of North Carolina at Chapel Hill, Chapel Hill, North Carolina, USA, unc.edu; ^7^ Integrated Well-Being Program, UNC Health, Chapel Hill, North Carolina, USA, unc.edu

## Abstract

**Background:**

Burnout assessments yield results of up to 65% for all emergency healthcare professionals and 53% for emergency nurses. We conducted a mixed methods study using a convergent parallel approach and a multilevel design with emergency nurses and other healthcare professionals to identify workplace stressors associated with burnout and prioritize those stressors for improvement.

**Materials and Methods:**

Emergency healthcare professionals were recruited from the emergency department of a large acute care hospital. Participants completed the abbreviated Maslach Burnout Inventory and a workplace stressor survey to contribute an overview of the current state of burnout and stress, participated in focus groups to provide context for identified stressors, and consented to contextual inquiries to nuanced in situ observation of stressors. From the survey, focus group, and contextual inquiry data, we employed a thematic analysis to develop an affinity model. Emergency healthcare professionals validated the stressors in the affinity model, prioritized potential improvement efforts, and rated the impact of and effort required for those improvements.

**Results:**

The participant sample was diverse in age, race, sex, shift time, and length of employment. Eighty‐one percent of the 63 survey participants met the criteria for burnout. Six focus groups with 19 participants and 22 contextual inquiries identified key work stressors related to staffing levels, workflow, communication, and care coordination. Using the affinity model, participants prioritized workflows, patient safety, patient‐related stressors, physical work environment, staffing, extrinsic motivation, technology, communication, and psychiatric patient procedures as areas for improvement.

**Conclusion:**

We successfully completed surveys, focus groups, and contextual inquiries with emergency nurses and other healthcare professionals to identify workplace stressors and prioritize those stressors for improvement efforts. This appears to be a viable method to formally identify and prioritize the unique stressors contributing to burnout and guide efforts to reduce burnout among emergency healthcare professionals.

## 1. Introduction

Burnout is a syndrome characterized by emotional exhaustion, depersonalization, and a low sense of personal accomplishment in the workplace resulting from a variety of work‐related stressors, psychosocial vulnerabilities, and coping styles [1]. The World Health Organization recognized burnout as a severe health issue, declaring it an “occupational phenomenon” in the International Classification of Diseases, 11th revision [2].

Although burnout affects all professions, burnout in healthcare is particularly widespread. The 2023 Veterans Health Administration survey [3] used the abbreviated Maslach Burnout Inventory to establish burnout rates of 27.6%–56.5% for all healthcare professionals (HCPs) and 31.4%–39.6% for nurses. Annual survey results from 2018 to 2023 exhibit consistent burnout rates before the COVID‐19 pandemic with a sharp increase during the pandemic and noticeable decrease after the pandemic [3]. However, rates have not returned to prepandemic levels [3]. If left unaddressed, burnout impacts HCPs’ physical and mental health, quality of patient care, and healthcare costs and exacerbates health disparities, among other issues [4].

Emergency HCPs are markedly affected, with reported burnout rates from 35% to 65% [5–8] among all staff and about 53% [9, 10] among nurses specifically. One study also found higher turnover and burnout among emergency department (ED) nurses than those working in other areas of medicine. The study attributed this difference in part to erratic work schedules, violence, and continual exposure to trauma [11]. As the ED is critical to our healthcare system, it is imperative to address emergency HCP burnout, particularly among nurses.

The 2019 National Academy of Medicine (NAM) report on HCP burnout includes a Systems Model of Clinician Burnout and Professional Well‐Being detailing contributing factors [1]. The report emphasizes that burnout is not a personal failing of HCPs but is a failing of the system in which HCPs practice. The NAM model’s system approach provides a framework for understanding how the healthcare system and society can interact with HCP characteristics to create an environment in which burnout is less likely. Workplace stressors in the model are classified into job demands (10 stressors) and job resources (11 stressors). Job demands require physical or psychological effort or skill. If job demands consistently exceed personal capacity, then burnout can result. Job resources support progress toward work goals. When job resources are low, particularly if job demands are high, then burnout is also more likely to occur. The report gives broad recommendations for reducing burnout, such as creating a positive work environment, but the steps that are appropriate to reduce burnout in one organization or unit may not be the steps appropriate for another.

To improve processes, workflows, and culture in the healthcare system [4], the 2022 US Surgeon General’s advisory on HCP burnout recommends seeking HCP input but little research reflects the use of such an approach. To help fill that gap, we present a generalizable, flexible, and holistic method grounded in the NAM’s system model of burnout that incorporates HCP perspectives to effectively assess burnout and identify and prioritize the unique stressors associated with burnout in an organization. The goal of this study was to demonstrate the results of using this method in a large ED. Our objective is that external healthcare organizations can adapt this approach to meet their own needs.

## 2. Materials and Methods

### 2.1. Participants

Participants were emergency HCPs working in the ED of a large community hospital. The hospital has 665 inpatient beds and the ED employs 182 HCPs to care for over 60,000 patients per year in the Raleigh, NC area. We strove for a balance in diversity of demographics and professional characteristics (e.g., role [physicians, nurses, staff, advanced practice provider, paramedic, medical technicians, other/prefer not to disclose], years in position, age, and weekly hours). Recruitment methods are detailed per method in the next section.

### 2.2. Research Design

**FIGURE 1:**
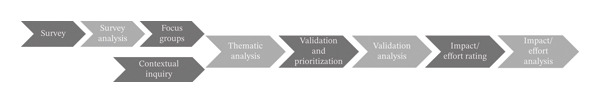
Study method flowchart for convergent parallel approach and multilevel design.

This study is oriented by Contextual Design [12], a type of system analysis, and the NAM’s systems model of burnout. We conducted a mixed methods study using a convergent parallel approach and a multilevel design (as shown in Figure 1) consisting of a survey, focus groups, and contextual inquiries to identify stressors contributing to burnout among emergency HCPs and synthesized the data into an affinity model. Emergency HCPs then validated the model, prioritized the stressors to address, and assessed the impact and effort of addressing each stressor. Emergency HCPs received protected time to complete study activities, and in‐person study activities were completed on site for convenience. The study protocol was reviewed and approved by the Institutional Review Board at the University of North Carolina at Chapel Hill (#20‐2359). Similar protocols have been used in other healthcare settings in prior studies [13–15] and iterated upon for use in this study. If an HCP expressed interest in further resources or we noticed a distressed participant, we referred them to the health system’s Well‐Being Program.

#### 2.2.1. Survey

All HCPs working in the ED were emailed an anonymous link for an online Qualtrics [16] survey (See Survey in Supporting Information A).

a. Demographics. Basic demographics including clinical role, years in position, weekly work hours, gender, race, and age.
b. Abbreviated Maslach Burnout Inventory [17, 18]. The scale is 0–6 with higher scores indicating higher emotional exhaustion or depersonalization. We chose the abbreviated version to minimize respondent burden.
c. Workplace stressor items. Twenty‐one items addressing the NAM model’s job demands and job resources were rated 1 to 5 for severity (1 = *Not at all*, 2 = *Low*, 3 = *Moderate*, 4 = High, 5 = *Extremely High*) and 1 to 4 for priority (1 = *Not an issue*, 2 = *Low Priority*, 3 = *Medium Priority*, 4 = *High Priority*). Anchors were provided for each rating but participants determined for themselves how they defined the severity or priority level for each anchor (ex., the severity level conveyed by “Moderate”). Each item also included a free‐text response box for optional comments or contextual information. Participants could define their own workplace stressors if desired.

#### 2.2.2. Focus Groups

The nurse manager and medical director informed all HCPs of focus group dates and times at least 2 days in advance via email. On the day of each focus group. The nurse manager, medical director, and research team also reminded HCPs via email and in person. At the beginning of each group session, we informed participants of study goals and obtained oral consent. Participants read and reflected on the survey’s top severity and top priority workplace stressors with free text responses before discussion with the goal of offering more detail on those stressors. We used open‐ended questions to elicit contextual information regarding experiences with each stressor rather than speculation about possible solutions. At the end, participants ranked those work stressors according to priority for improvement to assess whether the discussion had shifted their priorities. Sessions were audio recorded and transcribed with permission using otter.ai [19], and each transcript was reviewed and revised by two team members. To accommodate ED constraints, sessions lasted about 30 min (see Focus Group Guide under Supporting Information B).

#### 2.2.3. Contextual Inquiries

The team shadowed a representative sample of emergency HCPs recruited by the medical directors and nurse manager through purposive sampling considering role (RN and LPN), gender, race, length of tenure in role and at the organization, and other characteristics. These sessions involved the team observing HCPs in the course of their work in the ED, encouraging HCPs to describe their tasks, and asking HCPs questions when it was not disruptive. Researchers noted stressors and other information using field notes. Inquiries concluded with a 30‐min semistructured interview to further clarify events during the session. Sessions lasted between three and 6 hours. Team members entered notes into a common spreadsheet in Microsoft Excel [20] within 24 h of the inquiry. Notes were reviewed at weekly interpretation meetings to promote shared understanding and assess whether data saturation had been reached. Contextual inquiries were conducted until we reached data saturation where no new themes or insights were emerging (see Contextual Inquiry Guide in Supporting Information C).

#### 2.2.4. Validation and Prioritization

To ensure that the affinity diagram constructed from qualitative data (see Analysis below) represented the views of all emergency HCPs, we employed member checking to validate the data. All HCPs were informed of session dates and times via email and the nurse manager, medical director, and research team recruited in person on site prior to each session. The diagram was generated in Microsoft PowerPoint [21] and printed on a single sheet of paper. Participants reviewed the diagram and indicated stressors they agreed with and disagreed with, added amendments or annotations as they saw fit, and ranked their top five priorities for improvement.

#### 2.2.5. Impact Effort Rating

Using a technique from Lean Six Sigma [22], the themes prioritized during validation were compiled and synthesized into a Qualtrics [16] survey to evaluate the anticipated impact value and the level of effort needed to implement projects to address burnout. An electronic survey listing the prioritized themes with example stressors was distributed to all HCPs through email. HCPs subjectively rated each theme as high, medium, or low based on (a) the level of impact if addressed and (b) the level of effort needed to address the theme (see Impact Effort Survey in Supporting Information D).

### 2.3. Analysis

To maintain the participants’ voice throughout the analysis, as delineated by the Contextual Design [12], we integrated quantitative data with qualitative data and contextualized quantitative data with qualitative data through each step of the analysis as described below. This participant‐centered approach succeeds in providing objective results through descriptive statistics and qualitative context through a thematic analysis.

#### 2.3.1. Survey Analysis (Qualitative and Quantitative)

Quantitative analyses were completed in Microsoft Excel [20]. We calculated each participant’s burnout score using published criteria [17, 18] and summary statistics for all burnout scores. We calculated the mean rating scores and summary statistics for each stressor item and ranked the stressors by reported severity and priority. We compiled qualitative feedback for later thematic analysis. Quantitative results were used to rank stressors for the focus group presentation, while qualitative results gave context to the ranked stressors and provided a starting place for focus group discussions.

#### 2.3.2. Thematic Analysis (Qualitative)

We employed the thematic analysis to understand participant experiences. This inductive qualitative analysis method involved the team meticulously reviewing survey responses, focus group transcripts, and contextual inquiry notes to identify stressors then iteratively organizing them into key themes and subthemes that captured the essence of the participants’ perspectives on workplace stressors. Stressors are iteratively organized under the themes and subthemes until consensus is reached on the final structure and groupings. The themes and subthemes with supporting stressors comprise an affinity model [12] used during validation and prioritization sessions.

#### 2.3.3. Validation and Prioritization Analysis (Quantitative)

We evaluated the level of agreement for each affinity diagram item by dividing the number of agreements by the total number of agreements and disagreements for each item. The mean level of agreement was calculated as an average of all item agreement levels. Prioritized items were grouped by theme or subtheme for inclusion in the impact/effort survey and rank ordered.

#### 2.3.4. Impact‐Effort Analysis (Quantitative)

The mean of the impact and effort ratings were calculated per theme and plotted on a 2 × 2 matrix to visualize the relative ratings. In addition to the plot, the themes and their common stressors, as derived in Steps 2.3.2 and 2.3.3, were also listed by matrix quadrant with priority ranking to facilitate managers’ easy understanding of the highest priority stressors’ impact‐effort and priority ratings.

## 3. Results

### 3.1. Survey

The survey was emailed to 182 HCPs on 1 July 2022 and remained open through 22 July 2022. Sixty‐three HCPs completed the survey for a response rate of 35% (*n* = 63/182). Participants included 27 nurses, 16 physicians, and 20 other emergency HCPs (Table 1). Participants showed diverse representation across personal and professional characteristics except for gender and race where 66% of participants were female and 76% were White or Caucasian. Qualitative responses were compiled for use in the focus groups, and each individual stressor was itemized for later analysis.

**TABLE 1:** Survey participant demographics.

Demographics	*N* (%)	
Participants	*n* = 63/182 (35%)	

*Clinical role*		
Nurse	27/63	(43%)
Physician	16	(25%)
Staff	9	(14%)
Advanced practice provider	5	(8%)
Medical technician	2	(3.2%)
Paramedic	2	(3.2%)
Other/prefer not to disclose	2	(3.2%)

*Years in current position*		
< 1	12	(19%)
1‐2	8	(13%)
3‐4	9	(14%)
5–9	13	(21%)
10–14	4	(6%)
15–20	4	(6%)
> 20	11	(17%)
Prefer not to disclose	2	(3.2%)

*Age*		
< 25	6	(10%)
25–34	15	(24%)
35–44	13	(21%)
45–54	16	(25%)
55–64	6	(10%)
Prefer not to disclose	7	(11%)

*Usual weekly hours worked*		
< 40	34	(54%)
40–49	24	(38%)
> 50	5	(7.6%)

Participants’ mean level of emotional exhaustion was 3.46 (SD = 1.44), and their mean level of depersonalization was 2.91 (SD = 1.67), each on a scale of 1–6. Furthermore, 81% (*n* = 51/62, 95% CI [0.74, 0.88]) of respondents met criteria for burnout (defined as a score > 3) [18]. The rank order of stressor severity (Scale 1–5) and priority for improvement (scale 1–4) ratings were similar, with the top two items being inadequate staffing and inefficient workflows (Table 2).

**TABLE 2:** Workplace stressor severity and priority rankings.

Workplace stressor	Severity rank	Mean (SD)	Priority rank	Mean (SD)
Inadequate staffing^∗^	1	4.41 (1.02)	1	2.74 (0.48)
Inefficient workflows^∗^	2	3.92 (1.22)	2	2.58 (0.69)
Excessive workload^∗^	3	3.71 (1.01)	5	2.34 (0.67)
Time pressure^∗^	4	3.50 (1.17)	6	2.15 (0.78)
Patient‐related stressors^∗^	5	3.47 (1.02)	3	2.34 (0.68)
Interruptions and distractions^∗^	6	3.32 (1.28)	9	1.83 (0.84)
Physical work environment^∗∗^	7	3.03 (1.26)	4	2.20 (0.80)
Values and expectations alignment^∗∗^	8	2.83 (1.26)	8	1.84 (0.71)
Inadequate technology implementation^∗^	9	2.76 (1.15)	15	1.69 (0.78)
Extrinsic motivation and rewards^∗∗^	9	2.76 (1.22)	12	1.76 (0.77)
Lack of recognition for quality improvement activities^∗∗^	9	2.76 (1.28)	16	1.68 (0.80)
Organizational culture^∗∗^	12	2.74 (1.32)	7	1.87 (0.74)
Work‐life integration^∗∗^	13	2.64 (1.26)	10	1.83 (0.82)
Job control (flexibility and autonomy)^∗∗^	14	2.63 (1.19)	13	1.74 (0.77)
Administrative burden^∗^	15	2.53 (1.13)	20	1.59 (0.71)
Lack of dedicated time for professional development^∗∗^	16	2.47 (1.12)	19	1.59 (0.79)
Professional relationships^∗∗^	17	2.42 (1.16)	11	1.76 (0.79)
Moral distress^∗^	18	2.40 (1.11)	17	1.60 (0.68)
Intrinsic motivations and rewards^∗∗^	19	2.39 (1.11)	14	1.69 (0.72)
Unmanageable work schedules^∗^	20	2.27 (0.96)	18	1.60 (0.63)
Lack of support for research and teaching^∗∗^	21	2.02 (1.17)	21	1.52 (0.71)

^∗^Job demand.

^∗∗^Job resource.

### 3.2. Focus Groups

Focus groups were conducted 8–11 August 2022 by LV assisted by BS, JO, or EK, and participation was 10% (*n* = 19/182). Sessions were offered during weekdays, in the evening, and on weekends to give HCPs on all shift times an opportunity to participate. Due to space and time constraints, we could accommodate a maximum of 6 participants per session. We held six sessions with five in‐person and one via video conference. Participants per group ranged from one to six with two physicians, one physician assistant, eight nurses, five medical technicians, and three patient relations specialists. One session was attended by only one participant but due to recruiting constraints in a busy ED, we continued with the session using the same format and prompts as other sessions. To protect privacy due to the low number of participants, we tracked no other participant characteristics. Focus groups were transcribed, and each individual stressor was itemized for later analysis.

Participants provided context about key workplace stressors, particularly in the categories of inadequate staffing, inefficient workflows, patient‐related stressors, and time pressure. Many of these categories have an outsized effect on nursing staff.

• Inadequate staffing: The ED could use additional staff in all roles, but this was regarded as normal and they are accustomed to making do. However, the shortage of security and staff available to sit with psychiatric patients (known as “sitters”) is increasingly problematic. The presence of disruptive patients and visitors requires adequate security, and the current level of security is seen as inadequate to respond to these situations. Likewise, psychiatric patients with long ED wait times for placement require dedicated sitters to ensure safety for patients and staff. If sitters are unavailable, medical technicians or nurses are used, reducing clinical staff.
• Inefficient workflows: The workflow is seen as chaotic and unworkable, especially regarding use of the waiting area. Patients often experience long wait times, frequently move in and out of the waiting area, and are sometimes seen by emergency HCPs, particularly nurses, in the waiting area, raising concerns about operational efficiency and privacy.
• Patient‐related stressors: Disruptive patient and visitor behavior is not always handled effectively. Instead, emergency HCPs feel that this behavior contributes to the sense of an unsafe workplace where employees and their safety are not valued. This behavior was acknowledged to be directed at nurses more than other staff.
• Time pressure: The volume of patients and associated tasks, such as documentation, have increased, resulting in increased time pressure. This leads to less time for patient communications and makes it more challenging for emergency HCPs to do a thorough job.

Participants prioritized stressors after the focus group to allow us to determine if priorities shifted after the discussion. Table 3 shows that after the focus groups, inadequate staffing and inefficient workflows were still rated as top priorities.

**TABLE 3:** Workplace stressor priority rankings from the survey and after the focus groups.


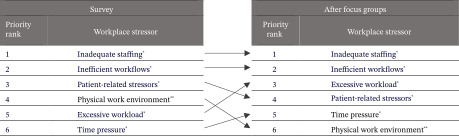

^∗^Job demand.

^∗∗^Job resource.

### 3.3. Contextual Inquiries

Contextual inquiries were conducted from 11 July to 18 August 2022 by LV, NB, EK, CCL, JO, and BS with 22 participants: seven physicians, five nurses, four medical technicians, three physician assistants, 2 patient relations specialists, and one health unit coordinator. To protect privacy, we tracked no other participant characteristics. Sessions were conducted during weekdays, in the evening, and on weekends to observe HCPs on all shift times. Emergency HCPs were observed for three to 5 hours per contextual inquiry while they went about their work. Total observation time was 77.5 h (mean = 3.5 h). Notes from contextual inquiries were compiled and discussed by the research team in weekly interpretation sessions during which the team reviewed the notes, itemized each stressor, and assessed data saturation.

### 3.4. Validation and Prioritization

During a four‐hour session, the team (LV, NB, EK, CCL, JO, and BS) derived themes and subthemes from the itemized stressors compiled from the survey, focus group, and contextual inquiry qualitative data. The themes and subthemes with supporting stressors were used to develop the affinity model. The resulting affinity model consists of 14 themes, 41 subthemes, and 300 stressors (Table 4). This model was used during validation and prioritization sessions.

**TABLE 4:** Number of items per workplace stressor theme and subtheme in the affinity model.

Stressor themes (*n*) (% agreement)	Stressor subthemes (*n*)
Workflows^∗^ (50) (82% agreement)	General (13) Triage/Zone 3 (13) Patient safety (9) Post‐triage (9 Discharge (6)

Organizational culture^∗∗^ (48) (66% agreement)	General (11) Management (10) Motivation (9) Communication (7) Coworker experience (5) Scheduling (4)

Physical work environment^∗∗^ (33) (92% agreement)	General (12) Patient beds in ED (8) Waiting area (8) Workspaces (3) Beds in hospital (2)

Patient‐related stressors^∗^ (30) (90% agreement)	Patient/family behavior (13) General (6) Time (4) Psychiatric patients (4) Nonemergency patients (3)

Workload^∗^ (24) (88% agreement)	General (14) Patient load (5) Self‐care (5)

Technology^∗^ (24) (55% agreement)	Epic (13) Communication (6) General (5)

Equipment and supplies^∗^ (18) (93% agreement)	Equipment and supplies (12) Computers (6)

Staffing^∗^ (16) (95% agreement)	General (12) Nursing (4)

Hospital factors^∗^ (14) (87% agreement)	Other departments (7) Laboratory (5) Radiology (2)

Interruptions and distractions^∗^ (11) (82% agreement)	General (11)

Policies and procedures^∗^ (10) (79% agreement)	General (5) Patients (5)

Documentation burden^∗^ (9) (80% agreement)	General (9)

Continuing education^∗∗^ (7) (79% agreement)	Professional development (5) Training (2)

Time pressure^∗^ (6) (90% agreement)	General (6)

^∗^Job demand.

^∗∗^Job resource.

Validation was conducted 20–22 September 2022 and participation was 5% (*n* = 9): four nurses, three physicians, one certified nursing assistant, and one medical technician. Four sessions were conducted by LV, assisted by BS or NB, on different days and at different times in a room adjacent to the ED. To protect privacy due to the low number of participants, we tracked no other characteristics. After validation, the affinity model was updated with the proportion of agreement for each stressor expressed as the number of participants agreeing divided by the total number of participants validating the stressor. Overall rate of agreement was 83% with consensus (100% agreement) achieved for 53% (*n* = 159/300) of stressors and some level of disagreement for the other 47% (*n* = 141/300). We are unable to share the model due to privacy restrictions but share the themes and subthemes, the number of stressors in each theme and subtheme, and the percent agreement per theme (Table 4).

Each participant listed their top 5 priorities for improvement from the stressors in the affinity model. The 9 participants listed a total of 40 priorities. We synthesized these items by nine themes and subthemes (Communication, Extrinsic motivation, Patient safety, Patient stressors, Physical work environment, Psychiatric procedures, Staffing, and Technology) to include in the impact‐effort rating survey.

### 3.5. Impact‐Effort Rating

**FIGURE 2:**
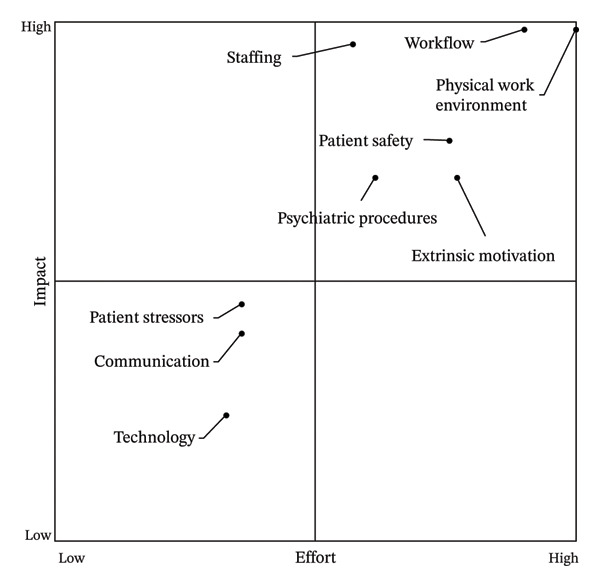
Plot of the mean perceived effort to address each theme against the mean perceived impact if each theme is addressed.

An impact‐effort rating survey for the 9 themes, including a description of common stressors, was disseminated via email to all emergency HCPs between October 25th and 31st, 2022, and completed by 80 participants (44.1% participation rate: 29 physicians and advanced practice providers and 51 nurses, medical technicians, and staff). For each theme, participants rated as High, Medium, or Low the amount of effort to address the theme and the amount of impact if the theme is addressed. The matrix plotting the mean effort and impact for each theme is in Figure 2 with themes and stressors listed below.

High Impact/High Effort themes and common stressors numbered by priority rank.

1. Workflow: improve waiting area utilization, triage process, and patient movement;
2. Physical work environment: improve boarding, layout, space utilization, and supply organization;
3. Extrinsic motivation: improve pay and vacation policies;
4. Patient safety: increase patient monitoring and reduce opportunities for error in workflows;
5. Staffing: hire additional sitters, nurses, technicians, and security;
6. Psychiatric patient procedures: improve therapeutic atmosphere, patient admission, and psychiatric patient placement processes.

Low Impact/Low Effort themes and common stressors numbered by priority rank.

1. Communication: improve communication with other departments and within the ED;
2. Technology: improve communication technology, ergonomics, and computer function;
3. Patient‐related stressors: introduce escalation procedures for problematic patient and visitor behavior.

## 4. Discussion

We used a mixed methods approach to assess burnout in a large acute care hospital ED and identify and prioritize workplace stressors through incorporation of the emergency HCPs’ perspectives. The study resulted in an emergency HCP‐driven ranking of nine stressor themes by priority and impact of and effort to address, including changes to the physical work environment, workflow, and staffing.

The initial assessment showed an 81% burnout rate among emergency HCPs, higher than the 35%–65% reported in other literature [5–8], along with a prioritization of workplace stressors to address. Many of the stressors aligned with dynamics affecting the hospital. First, the urban area around the hospital is growing quickly, possibly leading to increased patient volumes that stress the ED capacity. Second, disruptive patients and psychiatric patients are both resource intensive, and wait times for appropriate psychiatric care can be lengthy. Third, this study took place just after the COVID‐19 pandemic. Emergency HCPs were heavily impacted by the pandemic, and the toll was evident in their comments. Taken together, these factors place a cumulative strain on the ED and the people who work there and likely influenced the rate of burnout.

Details about these stressors and additional stressors, however, only became apparent with the results of the in‐depth participatory methods we employed. For example, the physical work environment is strained by the current ED volumes but also by the in‐patient admission workflows. Patients awaiting admission to the hospital often remain in ED beds and require nursing resources, reducing the ED’s ability to serve new patients. Next, the ED workflow is not efficiently handling high patient load. The triage area and the area where HCPs discuss results with patients are bottlenecks, further reducing the ability to serve new patients. Third, the communication technology is not suited to the often noisy and chaotic ED environment, causing frustration and inefficiencies when contacting personnel.

Overall, our results agree with the past findings concerning burnout in emergency HCPs [5–7, 11, 23] although the rate of burnout we found was higher than that in other studies. One study of emergency HCPs concluded that burnout was associated with being female, being a nurse, understaffing, and a desire to change the workplace [23]. Although it is important to remember that some factors are interrelated—for example, 90% of nurses are female and there is a long‐standing nursing shortage [24]—those results might partially explain the high burnout rate we observed because our sample was heavily comprised of nurses and females. Some other studies found that burnout was associated with an emotion‐oriented coping style [25], intolerance of uncertainty [26], and worse work‐life balance [27]. Our study did not assess coping style or tolerances, but work‐life balance was rated in the top half of stressors in the survey and was mentioned by participants in relation to other stressors such as stress from understaffing affecting relationships at home. A study of ED nurses in particular concluded that shift work and work schedule, workplace violence, and lack of management support were related to burnout [11]. Each of these topics was commented on during this study. Although HCP burnout has trended down since the pandemic [3] and this study’s burnout rate was higher than others, system factors influencing burnout are common and remain largely unresolved throughout the healthcare system. Burnout rates are likely to remain high unless systemic factors are addressed.

Though moral distress among HCPs is an area of growing awareness and study [28, 29], we noted that it ranked very low in our survey. However, it is woven through the comments we received during focus groups and contextual inquiries. For example, participants mentioned that patient volumes could result in leaving some patients in the waiting area who may have benefitted from closer monitoring. Although this discussion occurred in the context of inefficient workflow, moral distress is implicit in the concern that they are not serving the needs of patients as they would like. We categorized comments under the stressor category that participants referenced but stressor categories in the NAM model are not mutually exclusive. We are considering methods for representing this nuance in future work.

Results were compiled and presented to leadership. After viewing results, they indicated that although the results were much what they had expected, they now had data to back up their concerns when advocating for change. In the time since the study concluded, implemented improvements include new ED and psychiatric workflows, new waiting and triage area furniture to improve capacity, and staffing levels improvements. Future planned improvements include an ED expansion and additional security. Furthermore, informed by insights from this and similar projects, the health system is implementing a “Getting Rid of Stupid Stuff” (GROSS) [30] initiative to identify and eliminate unnecessary tasks that add burden without value. As part of the broader Well‐Being Program, GROSS focuses on streamlining workflows, reducing inefficiencies, and alleviating administrative burdens to ultimately support both staff well‐being.

Finally, published interventions, the NAM report, and the 2022 Surgeon General’s advisory give broad recommendations for how to address burnout in the healthcare system, but difficulties can arise when translating those recommendations into specific, concrete action items for a particular organization. The method presented here gives decision‐makers a blueprint for identifying and prioritizing organization‐specific stressors by incorporating contextual information only attainable through observation in the workplace and conversation with HCPs. We are unaware of these methods being applied to burnout in healthcare outside of our group [13–15] although they are widely applied in the human factors, usability, and design realms.

### 4.1. Limitations

The most important limitation is the relatively low participation rate that we saw during the validation phase despite protected time, long participation windows, reminders, and encouragement from department champions. We must note that his study was deployed in a busy ED with staff who were already under stress. However, the prioritized stressors were consistent throughout the course of the study, the participation in the impact effort rating was high, and we received no negative feedback on the priority items or suggestions for alternative priorities, giving us additional confidence that the results are representative. To obtain higher participation in future ED studies, we would recommend simply allowing more time for data collection to offset the limited time that ED HCPs can commit to participation. Second, a risk of bias in our results is attributable to the mix of people who participated. We recruited across a spectrum of characteristics and offered several modalities, days, and times for participation but could not control who chose to participate in surveys or validation sessions. We had more input in the makeup of focus groups and contextual inquiries where managers helped recruit a diverse set of participants to foster a balanced view. Again, the success of the impact effort phase may temper this source of bias. Finally, the study was conducted in a single ED in 2022. Although this may limit generalizability of our study results, the implementation confirmed that this method can be effectively deployed to produce customized improvement suggestions specific to any institution. The success of this study demonstrates the viability of the methods in other hospital settings.

## 5. Conclusion

We present a method for identifying and prioritizing stressors in a medical unit and show its application in a large ED. Using this method, we established a burnout rate of 81% among emergency nurses and other HCPs, gathered contextual information for factors contributing to burnout, and identified and prioritized the factors needing improvement. The top items were workflow, staffing, physical work environment, patient safety, and extrinsic motivation, and our contextual data will assist future improvement efforts. Each of these factors heavily impacts the work of nurses, and improvements will also heavily impact their work. Employing a similar approach, other healthcare organizations can identify and prioritize stressors contributing to burnout in their unique environment, make informed decisions about addressing stressors, and, in turn, help everyone in the organization providing better care to patients.

## Funding

The study was funded by UNC Health’s Well‐being Program and the National Institute for Occupational Safety and Health as part of the Centers of Excellence for Total Worker Health (grant number U19OH012303).

## Conflicts of Interest

Charul Haugan, Nadia E. Charguia are employed by UNC Health, the organization, providing part of the funding for the study. However, reported results were not influenced by this relationship.

## Supporting Information

Additional supporting information can be found online in the Supporting Information section.

## Supporting information


**Supporting Information** A: Burnout Survey. B: Focus Group Guide. C: Contextual Inquiry Guide. D: Impact Effort Rating Survey.

## Data Availability

Research data are not shared due to IRB restrictions.
