# Supplementary material for: Workplace Stressors Associated With Burnout Among Emergency Nurses and Other Emergency Healthcare Professionals: A Convergent Parallel Approach With a Multilevel Design
Source: J Nurs Manag. 2026 May 25;2026:7360364. doi: 10.1155/jonm/7360364 (PMC13201899; doi:10.1155/jonm/7360364)
Supplement: Supplementary file 1 — Supporting Information A: Burnout Survey. B: Focus Group Guide. C: Contextual Inquiry Guide. D: Impact Effort Rating Survey. [file JONM-2026-7360364-s001.docx]

**SUPPLEMENTARY MATERIAL A**

**BURNOUT SURVEY**

**INTRODUCTION AND CONSENT**

Thank you for agreeing to participate in this survey. The purpose of this survey is to gather information and develop an understanding of the factors contributing to healthcare professionals. The results will be used to guide targeted improvement efforts aimed at improving well-being and reducing workplace burdens.

**Your responses are confidential and will never be shared individually and will only be reported in aggregate.**

We appreciate you taking the time to provide your insights into the day-to-day life of a healthcare professional. There are a total of 5 pages and should take you between 15 and 20 minutes to complete.

**Note: We recommend taking this survey on a laptop / desktop for better view and ease of responding.**

Please click YES if you agree to participate and NO if you do not. You can review the full CONSENT FORM HERE.

⭘ YES

⭘ NO

**DEMOGRAPHICS**

**PART 1: Please answer the following questions related to demographics. We respect your privacy**. If you don't want to share any specific information, please use the *"Prefer not to disclose"* option.

How many years have you been in your current position?

⭘ < 1

⭘ 1 - 2

⭘ 3 - 4

⭘ 5 - 9

⭘ 10 - 14

⭘ 15 - 20

⭘ > 20

⭘ Prefer not to disclose

Typically, how many hours per week do you work?

⭘ < 40

⭘ 40 - 49

⭘ 50 - 59

⭘ 60 - 69

⭘ 70 - 79

⭘ > = 80

⭘ Prefer not to disclose

Which age group do you belong to?

⭘ <25

⭘ 25-34

⭘ 35-44

⭘ 45-54

⭘ 55-65

⭘ >=65

⭘ Prefer not to disclose

What best describes your gender?

⭘ Male

⭘ Female

⭘ Non-binary/third gender

⭘ Transgender Male

⭘ Transgender Female

⭘ Prefer to self-describe:___________________

⭘ Prefer not to disclose

Which of the following best represents your racial or ethnic heritage? Select all that apply.

🞏 White or Caucasian

🞏 Black or African American

🞏 Hispanic, Latino, Latina, or Latinx

🞏 Asian

🞏 American Indian or Alaska Native

🞏 Native Hawaiian or other Pacific Islander

🞏 Prefer to self-describe:_____________________

🞏 Prefer not to disclose

Which of the following best represents your marital status?

⭘ Single

⭘ Married

⭘ Divorced

⭘ Separated

⭘ Widowed

⭘ Prefer to self-describe:______________________

⭘ Prefer not to disclose

**PART 2: Please rate the following indicators of resilience and burnout.**

I am able to adapt when changes occur.

⭘ Not true at all

⭘ Rarely true

⭘ Sometimes true

⭘ Often true

⭘ Always true

I tend to bounce back after illness, injury, or other hardships.

⭘ Not true at all

⭘ Rarely true

⭘ Sometimes true

⭘ Often true

⭘ Always true

I feel burned out from my work.

⭘ Not true at all

⭘ Rarely true

⭘ Sometimes true

⭘ Often true

⭘ Always true

I have become more callous toward people since I took this job. *(Rate how often you generally feel this way.)*

⭘ A few times a year or less

⭘ Once a month or less

⭘ A few times a month

⭘ Once a week

⭘ A few times a week

⭘ Every day

**WORKPLACE FACTORS CURRENT STATUS**

**PART 3:** Please answer the following questions related to the factors that might be contributing to your workplace stress. *Please read the instructions before beginning this section*.

This page lists workplace factors that contribute to provider burnout and well-being (each row corresponds to a factor, listed in **bold** text). For each factor, representative examples have been provided for context.

**Your responses and comments on these factors will help us determine workplace improvement activities and guide follow-up focus groups.**

**INSTRUCTIONS**

For each factor, please provide your responses in the following columns:

1. **Current Status**: Please rate the degree to which these workplace factors have contributed to your stress over the last 3 months.
2. **Specific Examples**: Please use this space to indicate the specific issues / frustrations you experience related to this factor, especially when you rate *High* or *Extremely High.*

|  | **Current Status**  (Rate the degree to which these factors contribute to your workplace stress) | | | | | **Specific Examples** |
| --- | --- | --- | --- | --- | --- | --- |
|  | Not at all | Low | Moderate | High | Extremely High |  |
| **Excessive workload**  Amount of work to be performed exceeds the time in which to do the work; chronic high level of mental workload (divided attention, feeling rushed, information and communication overload); covering for infected colleagues. | ⭘ | ⭘ | ⭘ | ⭘ | ⭘ |  |
| **Unmanageable work schedules**  Excessive number of work hours on service, per week; Excessively long and continuous shifts (with post-shift overtime); amount of time / number of nights on-call per week; sleep deprivation | ⭘ | ⭘ | ⭘ | ⭘ | ⭘ |  |
| **Time pressure**  Constant, ever-present time pressure, feeling of chasing the clock; pressure to shorten time with patients; not enough time to complete clinical documentation during the workday. | ⭘ | ⭘ | ⭘ | ⭘ | ⭘ |  |
| **Inadequate staffing**  Perception of “short” staffing (too few support staff, too few providers / clinicians on service); chronic pressure to increase patient load. | ⭘ | ⭘ | ⭘ | ⭘ | ⭘ |  |
| **Inefficient workflows**  Inefficient, poorly defined, or variable workflows (ambiguous Standard Operating Procedures (SOP), variations between departments / units); inefficiently implemented workflows in Epic (e.g., med rec, medication orders); cancelled, missing, or delayed labs; documenting and tracking down others’ work (missing consults, recommendations, note bloat). | ⭘ | ⭘ | ⭘ | ⭘ | ⭘ |  |
| **Interruptions & distractions**  Excessive interruptions (paging for non-emergent issues); inappropriate default values in Epic; navigating changes due to Epic updates causes interruptions; patient portal messages and lab results; patient family questions outside the engagement time; excessive, poorly timed electronically generated messages; alerts and notifications; Vocera (wrong calls, loud volume) | ⭘ | ⭘ | ⭘ | ⭘ | ⭘ |  |
| **Inadequate technology implementation**  Epic; Vocera; pages; direct instant messaging; lack of integration between various applications within and outside Epic; lack of at-the-elbow IT support | ⭘ | ⭘ | ⭘ | ⭘ | ⭘ |  |
| **Moral distress**  Extending life beyond patient wishes / family pressure – treatment at all costs; rationing healthcare – who gets treated; high volume of COVID-related deaths; restriction of visitors; dying alone. | ⭘ | ⭘ | ⭘ | ⭘ | ⭘ |  |
| **Patient factors**  Verbally abusive patients / family; physically abusive patients / family; High-acuity patients require more clinical and non-clinical work. | ⭘ | ⭘ | ⭘ | ⭘ | ⭘ |  |
| **Lack of recognition for Quality Improvement activities**  Lack of dedicated time; lack of recognition for engagement in Quality Improvement activities. | ⭘ | ⭘ | ⭘ | ⭘ | ⭘ |  |
| **Lack of dedicated time for Professional Development requirements**  Lack of dedicated time for “necessary” professional development activities such as CME, re-certification, and Training: HIPAA, infection control, patient safety, etc. | ⭘ | ⭘ | ⭘ | ⭘ | ⭘ |  |
| **Administrative burden**  Excessive amount of time spent on administrative tasks (clinical (e.g., looking up labs) and non-clinical (e.g., billing)); administrative burden takes time away from meaningful clinical care (patient interaction). | ⭘ | ⭘ | ⭘ | ⭘ | ⭘ |  |
| **Lack of support for Research and Teaching**  Lack of protected time for research and teaching activities; lack of recognition for research activities and teaching activities. | ⭘ | ⭘ | ⭘ | ⭘ | ⭘ |  |
| **Professional relationships**  Poor interpersonal relationships with outside group colleagues / across disciplines; co-worker incivility, bullying, or abuse; lack of inter-relational coordination (communicating and relating to complete a task); lack of social support from administration; lack of collegial social support (peers); emotional contagion (feeling better / worse after interaction with positive / angry individual). | ⭘ | ⭘ | ⭘ | ⭘ | ⭘ |  |
| **Organizational culture**  Low leadership / management support; lack of leadership in setting tone for interprofessional conduct and collaboration; lack of fairness / equity in organizational policies and procedures. | ⭘ | ⭘ | ⭘ | ⭘ | ⭘ |  |
| **Physical work environment**  Unsatisfactory physical work environment; insufficient workspace; inadequate workstations / equipment; lack of adequate meeting space. | ⭘ | ⭘ | ⭘ | ⭘ | ⭘ |  |
| **Values and expectations alignment**  Inconsistent / unaligned values (unrealistic productivity measures vs. delivering high-quality excellent care); time spent on tasks / activities below license. | ⭘ | ⭘ | ⭘ | ⭘ | ⭘ |  |
| **Job control (flexibility and autonomy)**  Low sense of control over one’s practice environment; little autonomy over how one performs one’s work; lack of decision-making authority (e.g., in care delivery); inflexible work schedule; lack of involvement in shared governance (not having an influence on institutional policy). | ⭘ | ⭘ | ⭘ | ⭘ | ⭘ |  |
| **Intrinsic motivations and rewards**  Threats to intrinsic motivations such as meaning, purpose and joy in work, intellectual stimulation, engagement, sense of helping / healing. | ⭘ | ⭘ | ⭘ | ⭘ | ⭘ |  |
| **Extrinsic motivations and rewards**  Threats to extrinsic motivations such as money, prestige, praise; lack of feeling appreciated (by supervisors, peers, patients); lack of feeling respected (by administration, peers, patients). | ⭘ | ⭘ | ⭘ | ⭘ | ⭘ |  |
| **Work-life integration**  Struggles with work-life integration; work-home conflict; childcare concerns; schedules do not allow sufficient time away from office; necessity to use Epic remotely from home after work hours (pajama time); on-call schedule disrupts dedicated personal time. | ⭘ | ⭘ | ⭘ | ⭘ | ⭘ |  |

**WORKPLACE FACTORS PRIORITIZATION**

**PART 3 (continued):** Please prioritize the workplace factors as they apply to you. This page lists the same workplace factors you saw on the previous screen. *Please read the instructions before beginning this section.*

**INSTRUCTIONS**

For each factor, please provide your responses in the following columns:

1. **Priority for Improvement**: Rate the priority you believe (name of organization) Emergency Department should take in addressing and improving this factor. The higher you rate the priority, the greater urgency / need for targeting this factor for improvement.
2. **Comments**: Please use this space to express and thoughts related to the prioritization of these factors, especially when you rate *High Priority*.

|  | **Priority for Improvement**  (More urgent factors should receive higher priority ratings) | | | | **Comments**  (Please provide…) |
| --- | --- | --- | --- | --- | --- |
|  | Not an issue | Low Priority | Medium Priority | High Priority |  |
| **Excessive workload**  Amount of work to be performed exceeds the time in which to do the work; chronic high level of mental workload (divided attention, feeling rushed, information and communication overload); covering for infected colleagues. | ⭘ | ⭘ | ⭘ | ⭘ |  |
| **Unmanageable work schedules**  Excessive number of work hours on service, per week; Excessively long and continuous shifts (with post-shift overtime); amount of time / number of nights on-call per week; sleep deprivation | ⭘ | ⭘ | ⭘ | ⭘ |  |
| **Time pressure**  Constant, ever-present time pressure, feeling of chasing the clock; pressure to shorten time with patients; not enough time to complete clinical documentation during the workday. | ⭘ | ⭘ | ⭘ | ⭘ |  |
| **Inadequate staffing**  Perception of “short” staffing (too few support staff, too few providers / clinicians on service); chronic pressure to increase patient load. | ⭘ | ⭘ | ⭘ | ⭘ |  |
| **Inefficient workflows**  Inefficient, poorly defined, or variable workflows (ambiguous Standard Operating Procedures (SOP), variations between departments / units); inefficiently implemented workflows in Epic (e.g., med rec, medication orders); cancelled, missing, or delayed labs; documenting and tracking down others’ work (missing consults, recommendations, note bloat). | ⭘ | ⭘ | ⭘ | ⭘ |  |
| **Interruptions & distractions**  Excessive interruptions (paging for non-emergent issues); inappropriate default values in Epic; navigating changes due to Epic updates causes interruptions; patient portal messages and lab results; patient family questions outside the engagement time; excessive, poorly timed electronically generated messages; alerts and notifications; communication technology (wrong calls, loud volume) | ⭘ | ⭘ | ⭘ | ⭘ |  |
| **Inadequate technology implementation**  Epic; communication technology; pages; direct instant messaging; lack of integration between various applications within and outside Epic; lack of at-the-elbow IT support | ⭘ | ⭘ | ⭘ | ⭘ |  |
| **Moral distress**  Extending life beyond patient wishes / family pressure – treatment at all costs; rationing healthcare – who gets treated; high volume of COVID-related deaths; restriction of visitors; dying alone. | ⭘ | ⭘ | ⭘ | ⭘ |  |
| **Patient factors**  Verbally abusive patients / family; physically abusive patients / family; High-acuity patients require more clinical and non-clinical work. | ⭘ | ⭘ | ⭘ | ⭘ |  |
| **Lack of recognition for Quality Improvement activities**  Lack of dedicated time; lack of recognition for engagement in Quality Improvement activities. | ⭘ | ⭘ | ⭘ | ⭘ |  |
| **Lack of dedicated time for Professional Development requirements**  Lack of dedicated time for “necessary” professional development activities such as CME, re-certification, and Training: HIPAA, infection control, patient safety, etc. | ⭘ | ⭘ | ⭘ | ⭘ |  |
| **Administrative burden**  Excessive amount of time spent on administrative tasks (clinical (e.g., looking up labs) and non-clinical (e.g., billing)); administrative burden takes time away from meaningful clinical care (patient interaction). | ⭘ | ⭘ | ⭘ | ⭘ |  |
| **Lack of support for Research and Teaching**  Lack of protected time for research and teaching activities; lack of recognition for research activities and teaching activities. | ⭘ | ⭘ | ⭘ | ⭘ |  |
| **Professional relationships**  Poor interpersonal relationships with outside group colleagues / across disciplines; co-worker incivility, bullying, or abuse; lack of inter-relational coordination (communicating and relating to complete a task); lack of social support from administration; lack of collegial social support (peers); emotional contagion (feeling better / worse after interaction with positive / angry individual). | ⭘ | ⭘ | ⭘ | ⭘ |  |
| **Organizational culture**  Low leadership / management support; lack of leadership in setting tone for interprofessional conduct and collaboration; lack of fairness / equity in organizational policies and procedures. | ⭘ | ⭘ | ⭘ | ⭘ |  |
| **Physical work environment**  Unsatisfactory physical work environment; insufficient workspace; inadequate workstations / equipment; lack of adequate meeting space. | ⭘ | ⭘ | ⭘ | ⭘ |  |
| **Values and expectations alignment**  Inconsistent / unaligned values (unrealistic productivity measures vs. delivering high-quality excellent care); time spent on tasks / activities below license. | ⭘ | ⭘ | ⭘ | ⭘ |  |
| **Job control (flexibility and autonomy)**  Low sense of control over one’s practice environment; little autonomy over how one performs one’s work; lack of decision-making authority (e.g., in care delivery); inflexible work schedule; lack of involvement in shared governance (not having an influence on institutional policy). | ⭘ | ⭘ | ⭘ | ⭘ |  |
| **Intrinsic motivations and rewards**  Threats to intrinsic motivations such as meaning, purpose and joy in work, intellectual stimulation, engagement, sense of helping / healing. | ⭘ | ⭘ | ⭘ | ⭘ |  |
| **Extrinsic motivations and rewards**  Threats to extrinsic motivations such as money, prestige, praise; lack of feeling appreciated (by supervisors, peers, patients); lack of feeling respected (by administration, peers, patients). | ⭘ | ⭘ | ⭘ | ⭘ |  |
| **Work-life integration**  Struggles with work-life integration; work-home conflict; childcare concerns; schedules do not allow sufficient time away from office; necessity to use Epic remotely from home after work hours (pajama time); on-call schedule disrupts dedicated personal time. | ⭘ | ⭘ | ⭘ | ⭘ |  |

**ADDITIONAL WORKPLACE STRESSORS**

**PART 4**: Please share any ADDITIONAL FACTORS which you feel were not covered in the previous section but are important and need to be highlighted. *Please read the instructions before beginning this section.*

**INSTRUCTIONS**

For any additional factors that you mention in this section, please provide your responses (ratings and priority), as you did for the previous factors.

| List in the spaces below any other factors specific to your work that you find frustrating, broken, annoying, or burdensome. | **Current Status**  (Rate the degree to which these factors contribute to your workplace stress) | | | | | **Priority for Improvement**  (More urgent factors should receive higher priority ratings) | | | |
| --- | --- | --- | --- | --- | --- | --- | --- | --- | --- |
|  | Not at all | Low | Moderate | High | Extremely High | Not an issue | Low Priority | Medium Priority | High Priority |
|  | ⭘ | ⭘ | ⭘ | ⭘ | ⭘ | ⭘ | ⭘ | ⭘ | ⭘ |
|  | ⭘ | ⭘ | ⭘ | ⭘ | ⭘ | ⭘ | ⭘ | ⭘ | ⭘ |
|  | ⭘ | ⭘ | ⭘ | ⭘ | ⭘ | ⭘ | ⭘ | ⭘ | ⭘ |
|  | ⭘ | ⭘ | ⭘ | ⭘ | ⭘ | ⭘ | ⭘ | ⭘ | ⭘ |
|  | ⭘ | ⭘ | ⭘ | ⭘ | ⭘ | ⭘ | ⭘ | ⭘ | ⭘ |

**ADDITIONAL COMMENTS**

Please use this space to share any other thoughts, feedback, or concerns.

|  |
| --- |

**SUPPLEMENTARY MATERIAL B**

**FOCUS GROUP GUIDE**

**** Ensure Focus Group slides are pulled up on the screen before starting ****

Hi all, thank you for joining us today for our Well-being Deep Dive Focus Group. My name is [NAME] and I will be moderating our focus group session today. If at any point during our hour together you need assistance with anything or have a question, please don’t hesitate to ask.

With your consent, we will be recording today’s focus group. The recorded portion will be for research purposes only and will not be shared outside of myself and the research team. Do you consent to being recorded? **** Wait for response **** Thank you.

**** Start recording; go to slide 2, Agenda ****

Agenda

The purpose of this focus group is to get your thoughts, insights, and contextual information on system-related factors contributing to healthcare provider burnout. We don’t want you to solve the problems. Rather, we would like you to provide more details on what factors are contributing to your burnout.

During our time today, we will be:

- Viewing the quantitative data from the survey to identify top workplace stressors;
- Reviewing text responses on top stressors and providing contextual information and details; and
- Ranking which workplace stressors are your highest priority to address.

I will be summarizing the survey results based on the individual Qualtrics survey you filled out, and outlining what factors were rated as the most severe. These will include qualitative data in the form of text responses based on the top-rated factors on the surveys. As mentioned on the email with the survey link, text responses are anonymous.

**** Move to Survey Results slides ****

Survey Results

The survey was sent to [#] [role(s)] at [Division], with [X] completing the survey as of [day or date] for a completion rate of [Y%].

**** Move to Workplace Stressors slides ****

Workplace Stressors: Rating and Priority Ranks

Workplace stressors were rated in terms of severity and priority. This slide shows the aggregated data from the survey results and the top workplace stressors in terms of severity, and by priority. We will specifically be looking at data today that have the highest averages in terms of severity.

The next slide gives a closer look at the top workplace stressors by severity, with [Stressor 1] and [Stressor 2] tied as the stressors with scores of [X] and [Y] on a 5-point Likert scale.

[What are your thoughts on the results? Do you agree with them? Are you surprised by them?]

The next slide provides more detailed data on those top stressors by severity, with averages, range, and standard deviations provided. Next, we will be looking at text responses from the survey for these top stressors. There will not be any identifying information on the slides.

**** Move to quotes slides ****

Workplace Stressors: Text Responses

For each workplace factor’s text responses, we would like you to read through them on the screen and provide any contextual information or specific details to elaborate on the responses. This will allow us to get a more holistic and comprehensive view of the top workplace stressors.

The first top workplace stressor we will be starting with is [Stressor 1]. Please take the next couple minutes or so to read through some of the quotes and let me know your thoughts on the information on the slide.

**** Pause while they read. Wait for responses; if the conversation isn’t going, ask pointed questions below: ****

What are your thoughts on the quotes? Do you agree with them? Disagree with them?

What about this specific quote here? Could you tell me more about that?

**** Once sufficient time has been spent on the slide [~5-8 minutes], move to next slide ****

The next top workplace stressor we will be looking at quotes for is [Stressor 2]. Please take the next couple minutes or so to read through some of the quotes and let me know your thoughts on the information on the slide.

**** Repeat questions & process above until you get through all the text responses, OR you only have 5-10 min left ****

**** Move to Priority Scores slide ****

Prioritizations

Thank you for providing your thoughtful and honest responses to the text responses. Now that we have reviewed the qualitative data from the top workplace stressors by severity, we will next look at prioritizations on what you feel should be prioritized and addressed for improvement. These Priority Scores from the survey show [Stressor 1] was rated as the top workplace stressor to be addressed.

[What are your thoughts on the results? Do you agree with them? Are you surprised by them?]

The next slide has a more detailed look into the top workplace stressors with the highest priorities by average, with range and standard deviation also provided.

Prioritization Poll

Finally, the last portion of our focus group today will be a brief, one-question Prioritization poll. (VIRTUAL: **** Put Qualtrics poll link in chat. **** I just put a quick Qualtrics link in the chat.)

(IN PERSON: I am passing out a handout with the question.) Now that we have taken the time to discuss top workplace stressors, please take the next 1 minute or so to rank them by your individual priority to be addressed. This will help us throughout our process and will help focus future recommendations that will be provided to system leadership.

Wrap Up

Thank you for taking the time to participate in our well-being deep dive focus group.

(VIRTUAL: If you have not already completed it yet, please make sure to complete the Qualtrics Well-being survey, which will help inform us on individual thoughts and priorities regarding the stressors we talked about today and more.)

(IN PERSON: If you have not already, please return the handout with the prioritization question which will help inform us on individual thoughts and priorities regarding the stressors we talked about today and more.)

That concludes our well-being deep dive focus group. I will stick around if you have any questions. Otherwise, please have a great rest of your day!

**** End recording ****

**SUPPLEMENTARY MATERIAL C**

**CONTEXTUAL INQUIRY INSTRUCTIONS AND GUIDE**

**Checklist for the Observer**

- Introduce research team member(s)
- Complete introduction
- Complete oral consent
- Ask if there are any questions before beginning
- Observe and note items of interest within participant’s work environment
- Conduct interview
- Ask follow-up questions
- Thank participant and ask wrap-up questions

**Introduction**

Thank you for your time today to participate in the contextual inquiry. The main focus of our session today is to **understand the workplace stressors, how they result in frustration or breakdowns to your workflow, and how they contribute to burnout**. The goal is to be able to identify the workplace stressors contributing to healthcare professionals’ burnout and how to mitigate factors causing burnout. The session will take 3-4 hours.

We consider you **the expert** at your work so there are no wrong answers to any of our questions. While you answer questions or guide us through tasks, please focus on the details of how you **actually** do your work. It may help to think about the last time you performed the task and explain it to us as if we are going to need to perform the task just as you did. Please feel free to be honest and critical even if the way your work **actually** gets done is not the way you would **ideally like** for it to be done. We will be writing notes to record what is happening during these observations. You have the right to stop participating at any point. Everything you tell us and notes that we are writing will be strictly confidential.

**[obtain verbal consent]**

Any questions before we begin?

**CONTEXTUAL INQUIRY**

The CI will take approximately 3-4 hours while you unobtrusively observe the participant while they go about their work, asking questions when necessary, without interrupting the flow. The goal is to understand the work of the participant with a focus on what contributes to burnout, frustration, and breakdown in workflow. Before beginning, ask the participant to give a running commentary of what they are doing while completing a task. If they are silent at times while completing a task, prompt them to keep thinking aloud or ask follow-up questions during the interview portion if thinking aloud presents too much cognitive workload. Obtain permission from the participant before entering patient care areas. Ask that the participant introduce you and obtain permission before entering a private patient care area. During the CI, take note of:

- **Variables** – what are the demographics and behavioral variables?
- **Mental models** – how does the participant think about work, resources, services, and systems? What expectations do they have?
- **Activities** – what kinds of activities does the participant perform? Do they occur regularly or occasionally?
- **Motivation** – what are the participant’s goals? What does the participant enjoy doing?
- **Opportunities** – what are the participant’s pain points? What do they avoid?
- **Interactions** – who or what does the participant consult or work with?
- **Process** – how does the participant make decisions and perform tasks?
- **Sequences** – in what order is the participant performing tasks? Are there interruptions? Are they doing more than one thing at a time?
- **Tools** – what is the participant using to perform their work?
- **Problems** – what is frustrating or unexpected for the participant?

**INTERVIEW**

When the CI is done, conduct a short interview (approximately 30 minutes) to go over what you observed, ask questions you were unable to during the CI, and obtain details about topics you did not cover using the following questions:

- [Variables] How long have you been at [institution]?
- [Variables] How long have you been working at this location?
- [Variables] What is your primary role?
- [Variables] What are the different healthcare professionals with whom you work closely?
- [Variables] How do you communicate with other co-workers? What are the technology or tools that you use to communicate with other care team members and with patients? *How long have you used it? Have you used any others?*
- [Opportunities] What are the key workplace stressors? Why?
- [Activities] What do you do as a healthcare professional? Could you please describe a typical day for you at work? *Which activities occur regularly? Which activities occur only on occasion?*
- [Motivation] What do you enjoy most about your job as a healthcare professional? *Why? What activities do you always tackle first?*
- [Opportunities] What do you enjoy least about your job as a healthcare professional? *Why? What activities currently waste your time? What are the factors at your workplace that impede your performance?*
- [Activities] Can you tell us more about key stressors at your workplace? Can you help us understand what other factors contribute to workplace stress?
- [Variables] How many colleagues work with you closely? What are the different roles that you play as a care team?
- [Variables] How large is the care team? How often does it meet?
- [Interactions, Process] How do you work with your colleagues? What are the modes of communication with other team members? *What do you and your colleagues n=and co-workers do together?*
- [Activities, Mental Model] We’re interested in understanding physical, social, cultural, and psychological factors or activities or tasks contributing to burnout. Which of these types of activities do you actively participate in? How frequently do these activities take place?
- [Goals] What is/are the goal(s) behind these activities or tasks or factors?
- [Opportunities] Any frustrations with your current process of creating, deploying, collecting, or evaluating these activities? What frustrations do you have with this within?

**Wrap-up Questions**

- What did you think of this interview? *Anything you did not understand? Anything that made you anxious?*
- Is there anything else regarding the key workplace stressors contributing to healthcare professional burnout that you would like to add?
- Would you be willing to be contacted with follow-up questions?

**Thanks so much for your input. We really value your feedback. Have a great rest of your day!**

**SUPPLEMENTARY MATERIAL D**

**IMPACT / EFFORT RATING SURVEY**

Thank you for sharing your priorities during the Validation session. We would like you to organize the top priorities from that session against two variables:

- **Level of Impact** – how much value or impact the outcomes will have on the [*division*]
- **Level of Effort** – how much time, money, resources, and capacity will be needed to achieve the desired outcome.

Please click and drag each priority listed in the left-hand column to the most appropriate category box on the right. You can rank priorities as high impact or effort, medium impact or effort, or low impact or effort.

**EFFORT RATING**

| **Items**  **Physical work environment**: improve crowding, boarding, layout, space utilization, and equipment and supply organization  **Workflow**: introduce more efficient lobby utilization, triage process, and patient movement  **Staffing**: hire additional sitters, nurses, technicians, and security  **Patient safety**: increase monitoring and reduce opportunities for error in workflow  **Extrinsic motivation**: improve pay and vacation policies  **Psychiatric patient procedures**: improve therapeutic atmosphere, hold and placement processes  **Patient stressors**: introduce escalation procedures for poor patient and visitor behavior  **Communication**: address communication issues with other departments, such as laboratory and radiology, and within the ED to improve accountability and patient follow-up  **Technology**: address issues with communication technology, ergonomics, and computer function |  | **High Effort**  more cost, time, or resource intensive; more system involvement (ISD, HR, etc.); more co-workers needed to implement |
| --- | --- | --- |
|  |  |  |
|  |  |  |
|  |  | **Medium Effort** |
|  |  |  |
|  |  |  |
|  |  | **Low Effort**  less cost, time, or resource intensive; less system involvement; fewer coworkers needed to implement |
|  |  |  |

**IMPACT RATING**

| **Items**  **Physical work environment**: improve crowding, boarding, layout, space utilization, and equipment and supply organization  **Workflow**: introduce more efficient lobby utilization, triage process, and patient movement  **Staffing**: hire additional sitters, nurses, technicians, and security  **Patient safety**: increase monitoring and reduce opportunities for error in workflow  **Extrinsic motivation**: improve pay and vacation policies  **Psychiatric patient procedures**: improve therapeutic atmosphere, hold and placement processes  **Patient stressors**: introduce escalation procedures for poor patient and visitor behavior  **Communication**: address communication issues with other departments, such as laboratory and radiology, and within the ED to improve accountability and patient follow-up  **Technology**: address issues with communication technology, ergonomics, and computer function |  | **High Impact**  more long-term gain, more coworkers impacted, more metrics affected |
| --- | --- | --- |
|  |  |  |
|  |  |  |
|  |  | **Medium Impact** |
|  |  |  |
|  |  |  |
|  |  | **Low Impact**  fewer coworkers impacted, shorter-term impact, affects one group or metric |
|  |  |  |

Please share any other improvement ideas you have to support the [*division*]:

|  |
| --- |
